# Robotic Staging of Cervical Cancer With Simultaneous Detection of Primary Pelvic and Secondary Para-Aortic Sentinel Lymph Nodes: Reproducibility in a First Case Series

**DOI:** 10.3389/fsurg.2022.905083

**Published:** 2022-06-16

**Authors:** Philippe Van Trappen, Eveline De Cuypere, Nele Claes, Sarah Roels

**Affiliations:** ^1^ Department of Gynecology and Gynecological Oncology, AZ Sint-Jan Hospital Bruges, Bruges, Belgium; ^2^ Department of Medical Oncology, AZ Sint-Jan Hospital Bruges, Bruges, Belgium; ^3^ Department of Radiation Oncology, AZ Sint-Jan Hospital Bruges, Bruges, Belgium

**Keywords:** cervical cancer, indocyanine green, pelvic, para-aortic, robotic, sentinel lymph node, staging

## Abstract

**Objective:**

Discrepancies exist among international guidelines on the surgical staging of para-aortic lymph nodes in locally advanced cervical cancer (LACC), varying from considering a para-aortic lymph node dissection, at least up to the inferior mesenteric artery, to a complete para-aortic lymph node dissection. In this study, we aim to assess the reproducibility of our recently reported robotic technique using indocyanine green for identifying besides primary pelvic sentinel lymph nodes (SLNs), secondary para-aortic SLNs in a first case-cohort of cervical cancer patients.

**Methods:**

A retrospective case series of LACC patients with/without suspicious pelvic lymph nodes (LNs) on imaging (including two patients with an additional suspicious para-aortic LN) is reported. All patients underwent a robotic pelvic SLN and para-aortic sentinel/nonsentinel LN dissection using the da Vinci Xi platform. Indocyanine green was used as a fluorescent tracer, at a concentration of 1.9 mg/mL, and injected as 0.5 mL in each quadrant of the cervix.

**Results:**

In a total of 10 cases, primary pelvic SLNs (90% bilateral) with subsequent secondary para-aortic SLNs were identified in all cases. Lower para-aortic SLNs were present in all cases, and upper para-aortic SLNs were found in 9 out of 10 cases. The mean age of the cervical cancer patients was 49.8 years (SD ± 6.89), and the mean body mass index (BMI; kg/m^2^) was 23.96 (SD ± 4.60). The median total operative time was 105.5 min (range: 89–141 min). The mean numbers of primary pelvic SLNs and secondary lower and upper para-aortic SLNs were 3.10 (SD ± 1.10), 2.90 (SD ± 0.74), and 2.30 (SD ± 1.57), respectively. The median number of total para-aortic LNs (PALNs) dissected per patient was 11.5. Six patients had positive primary pelvic SLNs, and two had secondary positive para-aortic SLNs. The nonsentinel para-aortic LNs were negative in all cases. There were no intra- or postoperative complications.

**Conclusion:**

Our preliminary experience demonstrates the reproducibility of identifying, besides primary pelvic SLNs, secondary lower and upper para-aortic SLNs during robotic staging in LACC. A surgical approach limiting a complete para-aortic LN dissection could reduce the potential risks and morbidity associated with this procedure. To determine the sensitivity and negative predictive value of this new surgical approach, and whether the lower para-aortic SLNs under the inferior mesenteric artery are representative of the whole para-aortic region, large prospective observational studies are needed in LACC and/or those with suspicious pelvic LNs but apparent normal para-aortic LNs on imaging.

## Introduction

Cervical cancer is the fourth most common cancer in women worldwide, with globally an estimated 604,000 new cases and 342,000 deaths in 2020; more than 80% of these cases and deaths occur in low-to-middle-income countries (1). In the United States, cervical cancer accounts for 14,480 new cases and 4,290 deaths in 2021 (2). The 5-year overall survival rates range from 76.1% to 24% for locally advanced stages IB3 to IVA, according to the International Federation of Gynecology and Obstetrics (FIGO, 2018) (3).

Cervical cancer spreads in most cases first to the regional pelvic lymph nodes and subsequently to the para-aortic area, which is involved in 12%–25% of cases (4, 5). In addition to the evaluation of the pelvic lymph nodes (PELNs), the evaluation of the para-aortic lymph nodes (PALNs) is crucial and an important prognostic factor as well as guidance for both primary and adjuvant treatment (3, 5). Locally advanced cervical cancer (LACC) is treated by standard chemoradiation and extended para-aortic field radiotherapy is given in addition to patients with metastatic PALNs (6). Pretreatment imaging, including magnetic resonance imaging (MRI), computed tomography (CT), and ^18^F-FDG positron emission tomography-CT (PET-CT), is used to assess the PELNs and PALNs, but several studies have shown significant upstaging by pathology after surgical removal of these lymph nodes (LNs) (7, 8). Using only imaging to assess the retroperitoneal LNs may result in suboptimal staging in a significant proportion of patients with potential PALN metastases and undertreatment of the para-aortic area. Surgical staging, laparoscopic or robotic, of both PELNs and PALNs is therefore introduced but is related to potential increased operative risks and morbidity (5, 9, 10). Para-aortic surgical staging has been shown to contribute significantly to the individualization of radiation treatment of patients with LACC (11). Currently, discrepancies exist among international guidelines on the surgical staging of para-aortic lymph nodes in LACC, with an ongoing debate on surgical vs. radiological staging of the para-aortic region in LACC.

The European Society of Gynaecological Oncology (ESGO)/European Society for Radiotherapy and Oncology (ESTRO)/European Society of Pathology (ESP) guidelines advise that a para-aortic lymph node dissection, at least up to inferior mesenteric artery, may be considered in LACC with negative para-aortic lymph nodes on imaging, for staging purposes (12). On the other hand, the American National Comprehensive Cancer Network (NCCN) guidelines for cervical cancer recommend a complete para-aortic lymph node dissection for patients with LACC (13).

Sentinel lymph node (SLN) mapping in the pelvis has been introduced in cervical and endometrial cancer to reduce the potential morbidity related to a complete PELN dissection and is an accepted staging strategy for cervical and endometrial cancer surgery (14–17). Mainly, robotically assisted fluorescence imaging has been used recently to identify pelvic SLNs (PESLNs). Several reports exist on its application for identifying para-aortic SLNs (PASLNs) in endometrial cancer (18–24). The detection rate of PASLNs in these studies, mainly after cervical injection of indocyanine green (ICG), varies between 14% and 86%, and one report differentiates between lower and upper PASLNs (19). Although one recent large study showed rare locations of primary SLNs in cervical cancer patients such as in the para-aortic area, to our knowledge no studies to date have addressed the reproducibility of simultaneous detection of primary PESLNs and subsequent secondary PASLNs in cervical cancer (25). One recent video report showed para-aortic area nodes by using ICG during a laparoscopic PALN dissection (26). Our technique, using ICG for combined robotic pelvic and para-aortic sentinel node mapping in cervical cancer, has recently been published as a video report (27).

In this study, we report our initial experience, in a consecutive case series of cervical cancer patients with LACC with/without suspicious pelvic lymph nodes on imaging, using ICG for simultaneous detection of primary pelvic and secondary para-aortic sentinel lymph nodes during robotic staging.

## Materials and Methods

This is a single-center case series in a tertiary referral hospital in Belgium (Europe). Data were collected for cervical cancer patients undergoing a combined robotic pelvic and lower/upper para-aortic sentinel lymph node mapping using indocyanine green, as part of a standard pretreatment surgical staging, between April 2021 and February 2022. All medical (including operative and pathology) charts were reviewed after the first 10 cases. Patients included in the study had LACC with/without suspicious pelvic lymph nodes on imaging, magnetic resonance imaging (MRI) combined with computed tomography (CT), or 18F-FDG positron emission tomography-CT (PET-CT). Five out of ten patients had suspicious pelvic LNs on imaging, of which two patients had an additional suspicious para-aortic LN. Cervical cancer patients were finally staged according to the FIGO (International Federation of Gynecology and Obstetrics) classification of 2018.

All robotic surgical procedures were performed by only one qualified and experienced surgeon (gynecologic oncology surgeon), together with a resident. The robot da Vinci Xi platform (Intuitive Surgical inc., Sunnyvale, CA, USA) with near-infrared (NIR) fluorescence imaging (Firefly Technology) was used. Robotic instruments used were the fenestrated bipolar forceps, the Prograsp or Cadiere forceps, and the Vessel Sealer Extend or the SynchroSeal.

As a fluorescent agent, indocyanine green (ICG; Verdye 25 mg, Diagnostic Green GmbH, 85609 Aschheim-Dornach, Germany) was used at an optimized concentration of 1.9 mg/mL H_2_O. A total of 2 mL of ICG was used for each patient. After induction of anesthesia, all patients received a 0.5 mL injection of ICG, with a Microlance 21G needle, in each quadrant of the cervix, from superficial to deep. The needle was, after each injection, kept in place in the cervical stroma for a few seconds to avoid immediate spillage of fluorescent dye. After ICG injection, the four robot arms were docked after placement of the trocars. Subsequently, near-infra-red (NIR) fluorescence imaging was activated at the console. The surgical dissection of the pelvic sentinel lymph nodes (PESLNs) was initiated approximately 15 mins after cervical injection of ICG; the dissection of the PALNs (both sentinel and nonsentinel nodes), from the aortic bifurcation to the infrarenal area, was performed approximately 35–40 min after ICG injection. The robotic camera was rotated 180° when switching from a pelvic to a para-aortic LN dissection. All pelvic and aortic lymph nodes (sentinel and nonsentinel) were chronologically labeled during the operations, according to anatomical location. Subsequently, all pathological reports were reviewed and assessed in parallel, for anatomical location and sentinel/nonsentinel node, with the recorded videos of the operations. For all patients, the following data were recorded: age, body mass index (BMI; kg/m^2^), histological subtype, tumor grade, initial clinical FIGO-stage, radiological (r) and final pathological (p) FIGO-stage (2018), total and console operative time, estimated blood loss (EBL), intra- and postoperative complications, and length of stay (LOS) in the hospital. The numbers of PESLNs, lower PASLNs, upper PASLNs, total PALNs, positive PESLNs, and PASLNs were reviewed as mentioned above. As this is a retrospective noninterventional study, the Institutional Review Board (IRB) waived formal review and granted approval (IRB number: 2982). Basic descriptive statistics were used to calculate the mean, standard deviation, median, and range, in function whether the data were normally distributed or not.

## Results

**Figure 1:**
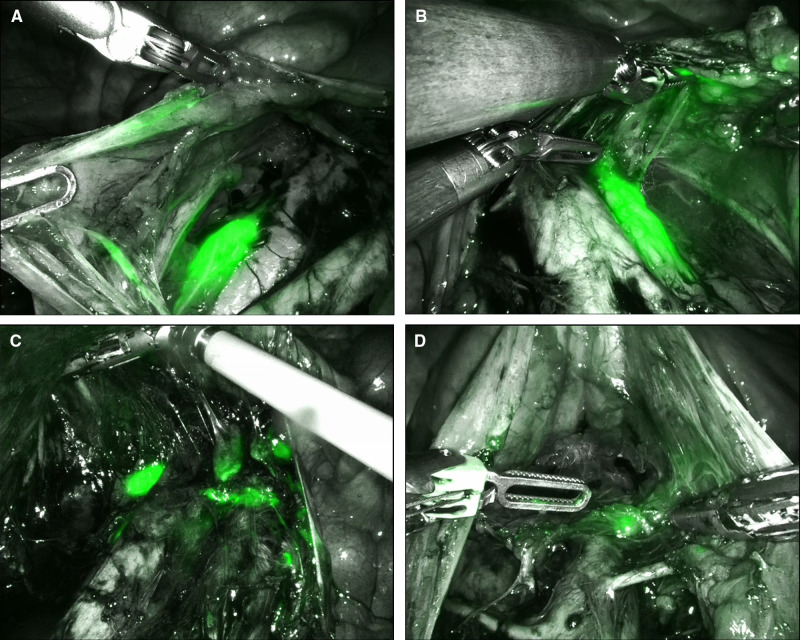
Lower and upper para-aortic sentinel lymph nodes (SLNs). (**A**) Secondary lower para-aortic SLN lateral from the inferior vena cava (IVC); (**B**): secondary lower para-aortic SLN lateral from the aorta; (**C**) tertiary upper para-aortic SLN lateral from the IVC; (**D**): tertiary upper para-aortic SLN above the inferior mesenteric artery in the infrarenal area.

During the period from April 2021 until February 2022, we collected data from the first 10 cervical cancer patients undergoing a combined robotic pelvic and lower/upper para-aortic sentinel lymph node mapping using indocyanine green, as part of a standard pretreatment surgical staging. All patients included had LACC with/without suspicious pelvic LNs on imaging (MRI combined with CT or PET-CT). Demographic and pathologic characteristics are summarized in Table 1. The mean age of the cervical cancer patients was 49.8 years (standard deviation (SD) ± 6.89), and the mean BMI (kg/m^2^) was 23.96 (SD ± 4.60). Four patients had as histological subtype a squamous cell carcinoma, five had an adenocarcinoma (one with a stratified mucin-producing intraepithelial type adenocarcinoma), and one had adenosquamous cell carcinoma. There were five grade 2 and five grade 3 tumors. Clinical FIGO-stage at presentation is as follows: three with 1B2, one with 1B3, one with 2A, and five with 2B (Table 1). According to the 2018 FIGO classification (including imaging) for cervical cancer, five cases were rFIGO-stage 3C1 (*n* = 3) or 3C2 (*n* = 2) based on imaging (Table 1). The median total operative time was 105.5 min (range: 89–141 min; Table 2). The median console time was 82.5 min (range: 62–116 min; Table 2). The median estimated blood loss (EBL) was 50 mL (range: 50–300 mL; Table 2). There were no intra- or postoperative complications related to the surgery. The mean length of stay (LOS) in the hospital was 2.2 days (SD ± 0.42). Table 3 depicts the surgical and pathological characteristics of the lymph nodes. Nine out of 10 cervical cancer patients had bilateral PESLNs. Lower and upper PASLNs were found in 100% and 90% of cases, respectively. The mean numbers of primary pelvic SLNs and secondary/tertiary lower and upper para-aortic SLNs were 3.10 (SD ± 1.10), 2.90 (SD ± 0.74), and 2.30 (SD ± 1.57), respectively (Table 4). The secondary lower PASLNs were in the inframesenteric area; the tertiary upper PASLNs were in the supramesenteric/infrarenal area (Figure 1A–D; supplementary material with video). The median number of total PALNs dissected per patient was 11.5 (Table 3). Four out of 10 patients were confirmed, after surgery, to have a pFIGO-stage 3C1 (*n* = 3) or pFIGO-stage 3C2 (*n* = 1), with micrometastasis or macrometastasis in PESLNs and/or PASLN (Table 3). The patient with pFIGO-stage 3C2 had, in addition to the macrometastatic para-aortic SLN (nonsentinel para-aortic LN negative), macrometastatic pelvic SLNs on both sides. Three out of 10 patients had isolated tumor cells (ITCs) in an SLN: two patients in a primary PESLN and one patient in a secondary PASLN without a positive primary pelvic SLN, and their initial FIGO-stage remained because ITCs are not included in FIGO-stage 3C (FIGO 2018). Nine out of 10 patients received primary chemoradiation therapy as well as brachytherapy, and one received extended field para-aortic radiotherapy (with PASLN macrometastasis). In all cases, the nonsentinel PALNs were negative. Of the three clinical FIGO-stage 1B2 patients, one was staged as rFIGO-stage 3C1 according to the 2018 FIGO classification and was also confirmed after surgery; one had a frozen pelvis due to severe endometriosis were a radical hysterectomy was technically not possible; both patients received primary chemoradiation therapy. The third patient with a FIGO-stage 1B2, with negative PESLNs and PASLNs (initially slightly enlarged pelvic LN on MRI but PET negative, and therefore limited PALN dissection in this case), underwent a subsequent radical hysterectomy.

**Table 1:** Clinical characteristics.

Patient	Age, year	BMI (kg/m^2^)	Histo type	Tumor grade	FIGO-stage (2018)
					cFIGO/rFIGO
1	49	27	Adeno	3	2B/3C2
2	40	21.3	Squamous	2	2B/3C1
3	51	21.7	Squamous	2	2A/2A
4	53	31.2	Adenosquamous	2	2B/3C2
5	46	24	Squamous	2	1B2/3C1
6	49	19	Adeno	3	1B2/1B2
7	63	20	Squamous	3	2B/3C1
8	57	19	Adeno	3	1B3/1B3
9	49	31	Adeno	3	1B2/1B2
10	41	25.4	Adeno	2	2B/2B

*FIGO, International Federation of Gynecology and Obstetrics; cFIGO, clinical FIGO stage;*

*rFIGO, FIGO including radiology;*

*adeno: adenocarcinoma;*

*Squamous,, squamous cell carcinoma;*

*adenosquamous, adenosquamous cell carcinoma.*

**Table 2:** Operative characteristics.

Patient	Total OT (min)	Console time (min)	EBL	LOS (days)
1	120	97	300	2
2	110	84	200	2
3	92	65	50	2
4	141	116	50	2
5	130	104	50	2
6	137	111	100	3
7	106	81	50	3
8	89	66	50	2
9	111	80	50	2
10	95	62	50	2

*OT, operative time; EBL, expected bloodloss; LOS, length of stay; Min, minutes.*

**Table 3:** Pelvic and para-aortic SLNs and pathology.

Patient	PeSLNs (L/R)	Lower PaSLNs	Upper PaSLNs	Total PaLNs	Pos PeSLNs	Pos PaSLNs	pFIGO-stage
	(*n*)	(*n*)	(*n*)	(*n*)	(*n*)	(*n*)	
1	2/1	2	2	5	3^**^	1^**^	3C2
2	1/2	4	2	18	2^**^	0	3C1
3	1/1	3	2	10	1^°^	0	2A
4	1/2	2	2	20	1^*^	0	3C1
5	2/3	3	5	16	1^*^	0	3C1
6	2/1	2	0	2	0	0	1B2
7	2/2	4	2	12	0	0	2B
8	2/2	3	5	14	1^°^	0	1B3
9	1/2	3	1	5	0	0	1B2
10	1/0	3	2	11	0	1°	2B

*L/R, left/right; n,number;*

*PeSLNs, pelvic sentinel lymph nodes; PaSLNs, para-aortic sentinel lymph nodes; PaLNs, para-aortic lymph nodes; Pos, positive;*

^°^ *,* *isolated tumor cells;*

^*^ *,* *micrometastases;*

^**^ *,* *macrometastases;*

*pFIGO-stage (2018), after surgery.*

**Table 4:** Summary of the mean number of pelvic and para-aortic sentinel lymph nodes.

	Pelvic SLNs	Lower para-aortic SLNs	Upper para-aortic SLNs
	(*n*)	(*n*)	(*n*)
Mean	3.10	2.90	2.30
Standard deviation (±)	1.10	0.74	1.57

*SLNs, sentinel lymph nodes.*

## Discussion

To the best of our knowledge, this is the first report on combined robotic pelvic and lower/upper para-aortic sentinel lymph node (SLN) mapping using indocyanine green (ICG) in locally advanced cervical cancer (LACC), with assessing its reproducibility in an initial case series.

The current FIGO-staging system allows the use of imaging to evaluate possible lymph node involvement in cervical cancer. A meta-analysis of the diagnostic performance of CT, MRI, and PET-CT for nodal involvement showed a sensitivity–specificity of 50%–92%, 56%–91%, and 82%–95%, respectively (3, 28). A systematic review of FDG PET-CT for nodal staging in cervical cancer in 8507 cases (with surgical pathology as reference) reported a pooled sensitivity of 72% (29). A recent meta-analysis, including seven cohorts with only patients with pelvic nodal metastases on imaging, but no suspicion of PALN involvement, revealed a pooled upstaging rate by PALN dissection of 21% (6). A complete negative PET or PET-CT upstaging by PALN dissection occurs in 12% of cases. As a consequence of suboptimal staging by imaging, a proportion of patients with unidentified PALN metastases may remain undertreated. On the other hand, a complete PALN dissection in all cases might lead to overtreatment and increased morbidity (5, 6, 8–10).

Discrepancies exist among international guidelines on the surgical staging of para-aortic lymph nodes in LACC. The ESGO/ESTRO/ESP guidelines advise that a PALN dissection, at least up to the inferior mesenteric artery, may be considered in LACC with negative PALNs on imaging, for staging purposes (12). On the other hand, the American NCCN guidelines for cervical cancer recommend a complete PALN dissection for patients with LACC (13). These guidelines highlight that in women with FIGO-stage IB to IIB cervical cancer, para-aortic LN involvement is closely related to the presence of pelvic LN metastases. The NCCN guidelines refer to data from 555 women who participated in GOG trials (GOG 85, GOG 120, and GOG 165). These GOG trials showed a more positive prognosis for patients who underwent a surgical dissection of PALN involvement vs. those who underwent radiological assessment of PALN involvement. On the other hand, the recently published uterus-11 trial showed no difference in disease-free survival (DFS) between surgical and radiological staging in LACC patients (30). However, there was a significant benefit in DFS for surgical staging in FIGO-stage 2B cervical cancer patients. Post-hoc analysis revealed also a cancer-specific survival benefit for the surgical (laparoscopic) staging group. They found a surgical upstaging in 33% of cases. A complementary editorial commented on this uterus-11 trial and concluded that a study is still needed with an adequate sample size, which is likely to show an improvement in survival for surgically staged patients (31).

Several reports exist on the application of sentinel node mapping, mainly by using ICG, for identifying para-aortic SLNs (PASLNs) in endometrial cancer (18–24). The detection rate of PASLNs in these studies varies between 14% and 86%, and one report differentiates between lower and upper PASLNs (19). This latter report could identify lower and upper PASLNs in 67.1% and 38.2%, respectively.

Geppert et al. described in endometrial cancer three distinctive lymphatic pathways from the pelvis towards the aortic basin, after injecting a fluorescent dye (ICG) into the cervix/uterine fundus: the upper paracervical pathway (UPP), the lower paracervical pathway (LPP), and the infundibulopelvic pathway (IPP) (32). The LPP goes via the internal iliac area to the medial site of the common iliac artery and then to the medial paraaortic/precaval LNs. The UPP goes via the uterine artery to the external/obturator area and subsequently lateral to the common iliac artery towards the lateral precaval/paraaortic LNs. Persson et al. described in 96% of endometrial cancer cases bilateral PESLNs, demonstrating a crucial lymphatic pathway, as described above, for tumor spread via the pelvis for uterine cancer (33). Although endometrial and cervical cancers are two distinctive different tumor types, they both arise from a different part of the same organ (uterus) with embryologically a mutual Mullerian origin and may therefore follow similar lymphatic pathways along the uterine vessels to higher up for lymphatic spread.

In our study, identifying simultaneously both primary PESLNs and secondary PASLNs in LACC patients, we found in 90% of cases bilateral primary PESLNs with subsequent secondary lower and upper PASLNs in 100% and 90% of cases, respectively.

Our median total operative time was 105.5 min, and this was considerably lower compared to most previous reports on robotic pelvic and para-aortic lymphadenectomy, with operative times varying between 85.4 and 283 min in the literature (34).

Given the description of the uterine lymphatic pathways by Geppert et al., it demonstrates that after injection of the fluorescent dye into the cervix, the PESLNs are the primary SLN stations (32). In our study here, the lower PASLNs seem to be the secondary SLN stations, as 90% of cases had primary and bilateral PESLNs, and tumor cells may spread along this cascade/lymphatic pathway. The importance of PESLNs as primary SLN stations in cervical cancer has also been shown recently in a large study by Balaya et al. *(*25)*.* They showed in 326 patients, with 1104 intraoperative detected SLNs, that the primary SLNs were mainly located in the pelvic (interiliac/external iliac) area in 83.2% of cases and only in 1.5% in the para-aortic area.

A surgical approach limiting a complete PALN dissection by using a combined primary pelvic and secondary para-aortic sentinel lymph node mapping technique, as reported here in our study, could reduce the potential risks and morbidity associated with this procedure, especially in the presence of increased surgical risk factors such as in obese and frail elderly patients (35). Similar to the primary pelvic sentinel lymph nodes, histologic ultrastaging using immunohistochemistry for identifying micrometastasis could be applied to the secondary para-aortic sentinel lymph nodes (36). A PALN dissection is especially indicated for patients with LACC and/or those with suspicious pelvic LNs but apparent normal para-aortic LNs on imaging. However, to establish the sensitivity and negative predictive value of this new surgical approach large prospective observational studies are needed. Similar to the pelvic sentinel lymph nodes (PESLNs), one need to evaluate whether the lower PASLNs (with histologic ultrastaging) under the inferior mesenteric artery are representative of the whole para-aortic area.

## Data Availability

The raw data supporting the conclusions of this article/Supplementary Material will be made available by the authors, without undue reservation.

## Supplementary Material

**Supplementary Video** | “Robotic staging in cervical cancer: real-time lymphatic spread of indocyanine green for identifying primary pelvic and secondary para-aortic sentinel lymph nodes”, related to this manuscript (case 10).
