# Supplementary material for: Robotic Staging of Cervical Cancer With Simultaneous Detection of Primary Pelvic and Secondary Para-Aortic Sentinel Lymph Nodes: Reproducibility in a First Case Series
Source: Front Surg. 2022 Jun 16;9:905083. doi: 10.3389/fsurg.2022.905083 (PMC9244622; doi:10.3389/fsurg.2022.905083)
Supplement: Supplementary Video | “Robotic staging in cervical cancer: real-time lymphatic spread of indocyanine green for identifying primary pelvic and secondary para-aortic sentinel lymph nodes”, related to this manuscript (case 10). [file Table_5_v1.docx]

**SUPPLEMENTARY MATERIAL.**

New Video, entitled “Robotic staging in cervical cancer: real-time lymphatic spread of indocyanine green for identifying primary pelvic and secondary para-aortic sentinel lymph nodes”, related to this manuscript (case 10). YouTube link: <https://youtu.be/cnyTBWjKoRE>
